# Waist Circumference and Mid−Upper Arm Circumference in Evaluation of Obesity in Children Aged Between 6 and 17 Years

**DOI:** 10.4274/jcrpe.v2i4.144

**Published:** 2010-12-10

**Authors:** M. Mümtaz Mazıcıoğlu, Nihal Hatipoğlu, Ahmet Öztürk, Betül Çiçek, H. Bahri Üstünbaş, Selim Kurtoğlu

**Affiliations:** 1 Department of Family Medicine, School of Medicine, Erciyes University, Kayseri, Turkey; 2 Departman of Pediatric Endocrinology, Sisli Etfal Education and Research Hospital, Istanbul, Turkey; 3 Department of Biostatistics, School of Medicine, Erciyes University, Kayseri, Turkey; 4 Department of Nutrition and Dietetics, Atatürk Health School, Erciyes University, Kayseri, Turkey; 5 Department of Pediatric Endocrinology, School of Medicine, Erciyes University, Kayseri, Turkey; +90 352 437 52 85+90 536 323 03 02nihalhatipoglu@yahoo.comDepartment of Pediatric Endocrinology, Sisli Etfal Education and Research Hospital, İstanbul, Turkey

## Abstract

**Objective**: The purpose of this study was to determine the cut−off values for waist circumference (WC) and mid−upper arm circumference (MUAC) and to assess their use in screening for obesity in children.

**Methods**: Anthropometric measurements of a total of 2621 boys and 2737 girls aged 6−17 years were analyzed. WC and MUAC values were compared with ROC analysis using body mass index (BMI) cut−off values of the International Obesity Task Force (IOTF) and using WC≥ 90th percentile.for MUAC.

**Results**: In both genders, except for boys and girls in the 6−year age group and post−pubertal boys, the differences between area under curve (AUC) values for WC and MUAC were not significant, indicating that both indices performed equally well in predicting obesity. Sensitivity was suboptimal through age groups 6−9 years in the boys and sensitivity was suboptimal at 6, 7,14 and 17 years both in boys and girls.

**Conclusions**: We conclude that MUAC can be a useful parameter in screening obesity and body fat distribution in children and, can be applied in epidemiological studies and in clinical practice.

**Conflict of interest:**None declared.

## INTRODUCTION

The global trend of increasing childhood obesity is well documented. Obesity in childhood has therefore become a health issue of concern to health professionals throughout the world as a leading factor for certain chronic diseases such as hyperlipidaemia, hyperinsulinemia, hypertension, and early atherosclerosis (1, 2, 3). Whether it persists or not in adulthood, childhood obesity is substantially related with increased morbidity and mortality (4). However, the detection of obesity during childhood is more difficult than during adulthood due to the developmental changes in children. Additionally, there is no general consensus on the reliability, use, application of direct and indirect anthropometric indices describing obesity in children (5).

For diagnosis of obesity and for evaluation of current and future metabolic risks, individual assessment with body mass index (BMI) is essential, but additional anthropometric indices are needed to describe accurately the body fat distribution. Although precise methods to determine body fat content and distribution exist, these methods are not practical for epidemiologic studies. On the other hand, anthropometric indices provide a valid tool to screen large groups (6).

Waist circumference (WC), skinfold thickness and mid−upper arm circumference (MUAC) are the leading indirect methods used to assess fat reserve and the application of these anthropometric indices is recommended to screen the child and adolescent population for obesity (7).

This study aimed to evaluate 1) the role of WC and MUAC, used in addition to BMI, in determining overweight and obesity, 2) to establish cut−off values for defining overweight and obesity with WC and MUAC (overweight and obesity are defined according to BMI).

## METHODS

The data analyzed in this study were based on measurements obtained in a study entitled “Determination of Anthropometric Measures of Turkish Children and Adolescents” (DAMTCA I) and conducted in the time period from February to April 2006 (8). Children and adolescents residing in Kayseri, Turkey constituted the study sample. Kayseri province is a leading industrial and trade centre, which has more than 1000 000 inhabitants and receives emigrants from different parts of Turkey. Of a total of 5727 primary and secondary school students included in the above−mentioned study, data regarding WC, skinfold thickness and MUAC were available in 2737 girls and 2621 boys, aged 6−17 years. Methodology relating to sample selection, weight and height measurements was given in the previous publication (8).

BMI (kg/m2) was calculated as weight (kg) divided by the square of the height (m2). WC and MUAC were measured to the nearest 0.1 cm with an anthropometric tape over light clothing. WC was measured at the minimum circumference between the iliac crest and the rib cage. MUAC measurements were taken in centimeters with non−elastic tape to the nearest 0.1 mm on the upper left arm (halfway between the acromion process and the olecranon process). The children/adolescent stood relaxed with his/her side to the trained technician and the arm hanging freely at the side; the tape was passed around the arm at the level of the mid−point of the upper arm.

Results were presented as the mean 95% confidence interval (95%CI), median, minimum−maximum (min−max) for each age and gender. Simple linear regression analyses (R2) were computed to explore the relationships between BMI, WC, and MUAC for each age.

The WC≥90th percentile values for age and gender were used to identify children and adolescents with abdominal obesity in accordance with the International Obesity Task Force (IOTF) cut−off values for overweight and obesity (9). The performance and cut−offs of anthropometric indices were determined by the receiver operating characteristic (ROC) analysis (10). The ROC curves demonstrated the overall discriminatory power of a diagnostic test: BMI, WC, and MUAC. The better test has a curve skewed closer to the upper left corner. The area under the ROC curve (AUC) is a measure of the diagnostic power of a test. The perfect test will have an AUC of 1.0, while an AUC value of 0.5 indicates that the test performs no better than expected by chance. Sensitivity and specificity of the anthropometric indices have been calculated at all possible cut−off points to find the optimal cut−off value. The optimal sensitivity and specificity were the values yielding maximum sums from the ROC curves (Clinical significance of ‘cut−off’s were checked with the Youden index). Cut−off values and AUCs of WC and MUAC were compared for each age and gender. MedCalc software was used to test the significance of the differences for the AUCs.

Agreement between these anthropometric indices were assessed by Cohen’s κ statistic, with values of 0.00 to 0.20 indicating poor, 0.21 to 0.40 − fair, 0.41 to 0.60− moderate, 0.61 to 0.80−good, and 0.81 to 1.00 − excellent concordance (11).

## RESULTS

The current study included 5358 subjects (2621 boys and 2737 girls). The mean and medians of WC and MUAC for each age and gender are shown in Table 1. We determined the WC cut−off values by relating WC and MUAC with BMI according to the IOTF cut−off points. Since we could not find cut−off values for MUAC in the relevant publications, we used WC≥90th percentile as the cut−off value for the ROC analysis.

The AUC, cut−off value, sensitivity, and specificity for each age and gender are shown in Tables 2, 3, 4, 5. The AUC, both for WC and MUAC, were statistically significant in both genders in the age groups 6−17 years. The differences between AUCs for WC and MUAC were not significant, indicating that both indices performed equally well in predicting normal, overweight, and obesity (except 15− and 16−year−old boys) in each gender in 6−17 years old children (Tables 2, 3).

The sensitivity of WC for 6−8 years old boys and the sensitivity of MUAC for 6−8 and 15 years old boys were estimated to be suboptimal for clinical use (Table 2). The sensitivity of WC for 6,7,14,17 years old girls and the sensitivity of MUAC for 6−7 years old girls were also found suboptimal for clinical use (Table 3). The R2 calculated for R2 showed that the values for BMI and WC were higher than those for BMI and MUAC (Table 6).

The agreement between the two approaches (WC≥90th, MUAC≥90th percentile) to define abdominal obesity was moderate (κ=0.56, κ=0.50; p<0.001, respectively for boys and girls).

**Table 1:**
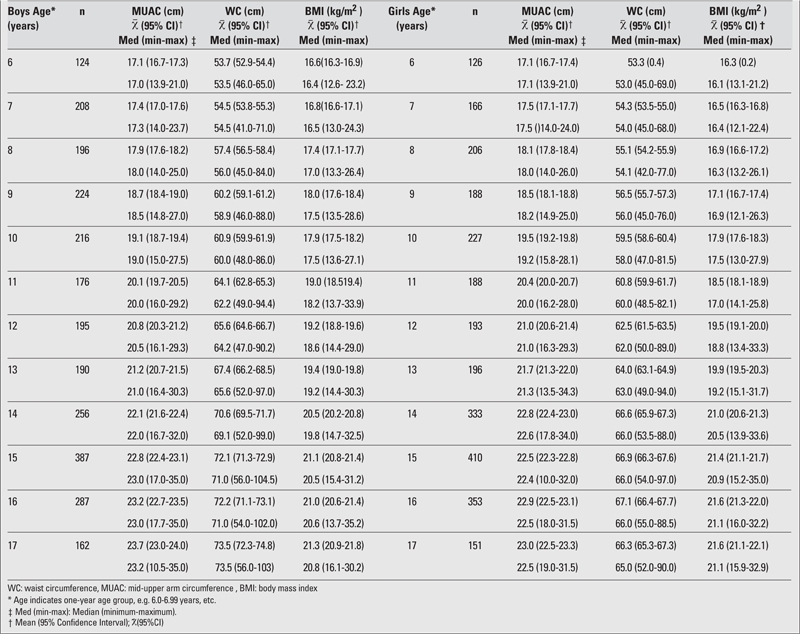
Mean (95%CI) and median (minimum−maximum) values for WC, MUAC, BMI in male and female Turkish children and adolescents

**Tables 2:**
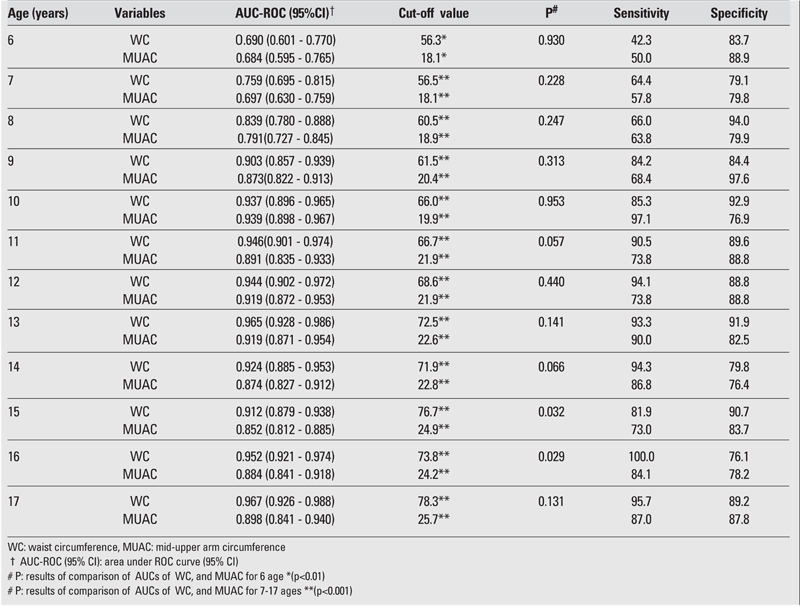
ROC curve analysis of WC and MUAC in male children and adolescents for overweight cut−off values

**3:**
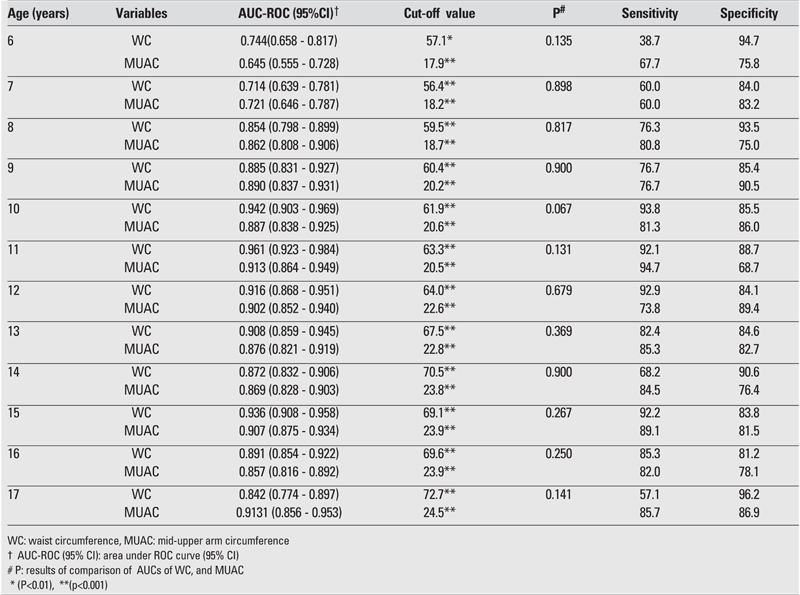
ROC curve analysis of WC and MUAC in female children and adolescents for overweight cut−off values

**4:**
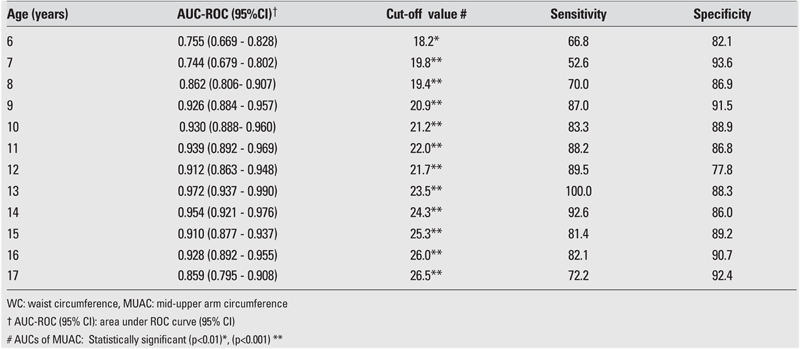
ROC curve analysis to determine cut−off MUAC values for WC≥ 90th percentile in male children and adolescents

**5:**
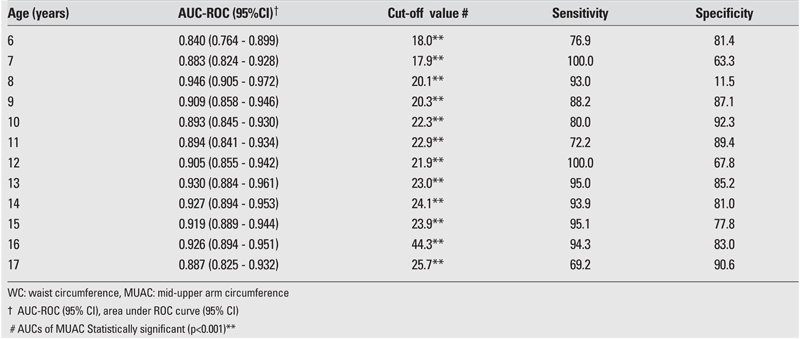
ROC curve analysis to determine cut−off values of MUAC for WC≥ 90th percentile in female children and adolescents

**Table 6:**
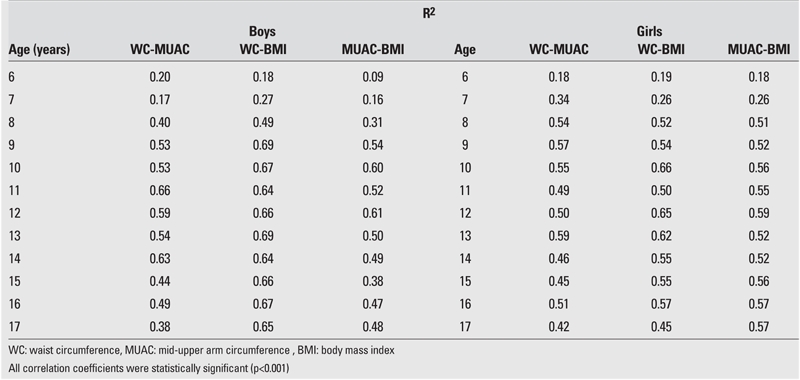
Simple linear regression coefficients (R2) between WC, MUAC, and BMI in male and female children and adolescents

## DISCUSSION

To the best of our knowledge this is the first and comprehensive study discussing the use of different anthropometric indices in evaluation of obesity in 6− to 17−yearold children.

Increasing obesity prevalence among children and adolescents is one of the leading public health problems globally. Simple and practical methods are needed in screening obesity. BMI is accepted as an index of body fat reserve, but for the same BMI, body fat reserve may be different between individuals. Another major drawback concerning BMI is that its measurement gives no indication of body fat distribution. It has been known for some time that a central distribution of body fat, particularly an excess accumulation of fat intraabdominally rather than a more peripheral distribution, carries a higher risk for obesity−related comorbidities. Hence, WC is proposed to describe body fat distribution as an index additional to BMI. Laboratory−based methods (e.g. dual−energy X−ray absorptiometry, underwater weighing) are also used to assess body fat in children, but these methods are expensive and usually limited to small−scale studies (12, 13).

In this study, we measured MUAC in addition to WC to describe obesity defined by BMI. We consider that each country must determine their own cut−off values for WC, BMI, and MUAC. While the use of BMI as a surrogate for fat excess among children raises debates, WC is increasingly recognized as a useful index reflective of both fat excess and risk of diseases (14). Some anthropometric indices like WC and MUAC, which are used to determine adiposity, show a good level of correlation with corporal mass (15).

Early identification and treatment of children with central adiposity is crucial to detect the risks for future metabolic complications. WC is considered as the best indicator of abdominal obesity, but in circumstances where WC measurement is not feasible (skeletal deformities, intraabdominal disorders or change in abdominal circumference related with respiratory movements), measurement of MUAC may be an alternative and reliable index. Thus, anthropometric ndicators such as BMI, WC and MUAC can be used as screening tools for obesity in children and adolescents (16, 17). However, systematic monitoring of WC and MUAC is not a commonly performed method in pediatric studies in many countries and internationally accepted cut−off values are also not yet established.

WC rather than BMI is recommended as an index of obesity−related health risks in adults (18, 19). WC is a highly sensitive and specific measure of truncal adiposity and a strong predictor of visceral adiposity also in the pediatric population. Furthermore, WC shows a relationship with the metabolic consequences of obesity, including negative lipid profile, increased blood pressure, and insulin resistance in children and adolescents (20). In adults, specific WC cut−off values are reported from different countries for screening metabolic syndrome, cardiovascular diseases, type 2 diabetes and hypertension, but studies describing specific WC cut−off values in children are scarce (21, 22, 23).

MUAC is proposed as another important indicator of obesity, and is also reported to closely reflect body fat tissue (24). Analyzing the NHANES data, Gortmaker and Dietz reported that while obesity prevalence was increased by 40% in a 20−year period, BMI values remained relatively constant (25). This finding indicates that the proportion of body fat and lean body mass have changed longitudinally.

Additionally, energy intake, growth, and fat storage characteristics of children may also lead to discordance in assessing overweight and obesity. Arm anthropometry appears as a popular, cheap and non−invasive method. Especially in epidemiologic studies, MUAC is a practical tool and can be measured easily almost in any situation. The primary limitation may be the absence of studies to determine the validity of this method (25).

Finally, we believe that the primary contribution of this present study was the finding that both WC and MUAC can be substituted for one another as an additional evaluation tool next to BMI in detecting overweight and obese children and adolescents. It was found that in boys, clinically significant WC cut−off values could be obtained at ages 9 to 17, while the optimal ages to obtain MUAC cut−off values were 9−14 and 16−17 years (Table 2). Optimal ages to get clinically significant WC and MUAC cut−offs in the girls were 8−13 years for WC and 15−16 years for MUAC.

In conclusion, our data revealed that both WC and MUAC show a good correlation with BMI and that these two parameters have the characteristic of indirectly defining the composition (lean and fat tissue content) of the body rather than providing information on total mass. We believe that the present study contributes to providing cut−off values for two practical tools, which can be used to determine body fat reserve. Additionally, these two indices may also be used in epidemiologic studies to assess cardiovascular and metabolic risk in overweight and obese children.

**Acknowledgments**

The authors would like to thank the students of Kayseri Health Institute for their valuable help in data collection and the Erciyes University School of Medicine for their support.

**Table 2:**
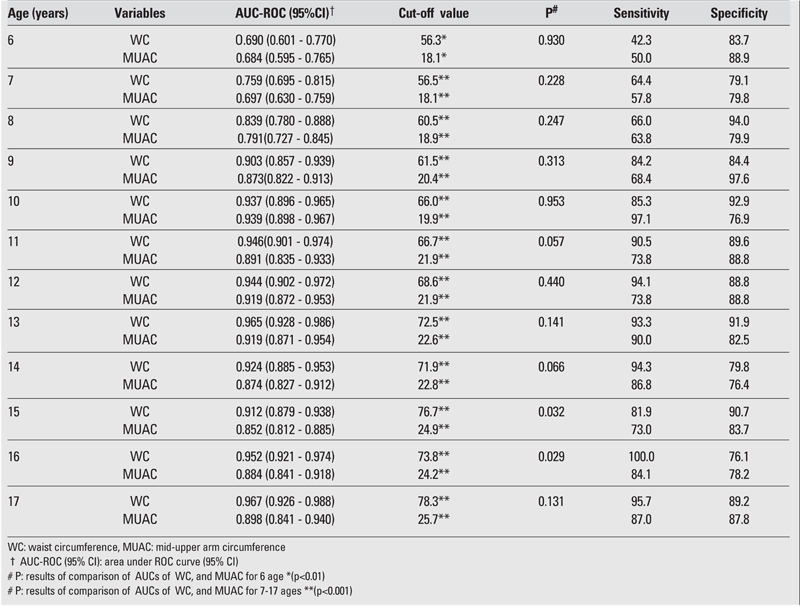
ROC curve analysis of WC and MUAC in male children and adolescents for overweight cut−off values
